# Relationship between the Prevalence of Ectoparasites and Associated Risk Factors in Free-Range Pigs in Kenya

**DOI:** 10.1155/2013/650890

**Published:** 2013-07-24

**Authors:** John Maina Kagira, Paul Njuki Kanyari, Ndicho Maingi, Samuel Maina Githigia, Chege Ng'ang'a, John Gachohi

**Affiliations:** ^1^Department of Animal Health and Production, Faculty of Agriculture, Jomo Kenyatta University of Agriculture and Technology, P.O. Box 62000-00200, Nairobi, Kenya; ^2^Department of Veterinary Pathology, Microbiology and Parasitology, Faculty of Veterinary Medicine, University of Nairobi, P.O. Box 29053, Nairobi, Kenya; ^3^International Livestock Research Institute (ILRI), Old Naivasha Road, P.O. Box 30709-00100, Kabete, Nairobi, Kenya

## Abstract

A cross-sectional study was undertaken to determine the prevalence of ectoparasites and possible risk factors in free-range pigs from 135 farms of Busia District, Kenya. Three hundred and six pigs were examined for presence of external parasites using standard parasitological methods. Data on management practices including housing and history of acaricide spraying were also collected. The ectoparasites found in the pigs were *Haematopinus suis* (96.1%), *Sarcoptes scabiei* (63.7%), and ticks (29.7%). The tick species included *Rhipicephalus appendiculatus* (70%), *Boophilus decoloratus* (31%), and *Amblyomma variegatum* (12%). The occurrence of the infestations was associated with age, being highest in sows (*S. scabiei*) and finishers (ticks and *H. suis*). Male pigs had highest prevalences of *H. suis* and ticks, while female pigs had highest prevalence of *S. scabiei*. The prevalence of the parasitic infestations was significantly (*P* < 0.05) associated with their origin being either lower (*H. suis* and *S. scabiei*) or higher (ticks) in pigs originating from divisions with high rainfall. Housed pigs had significantly (*P* < 0.05) lower prevalence of *H. suis* and ticks than those from households without pig housing. It is concluded that the free-range pigs have high prevalence of ectoparasites, and effective control strategies focussing on improved animal husbandry and acaricide use should be implemented.

## 1. Introduction

The main ectoparasites of pigs are *Sarcoptes scabiei var. suis* and *Haematopinus suis*. Sarcoptic mange, caused by *Sarcoptes scabiei var. suis*, is of major economic importance in pig farming as it significantly reduces production efficiency, and costs of acaricides used in its control are enormous and run into billions of dollars worldwide [1]. Although occurrence of sarcoptic mange is not widely studied in Kenya and African countries, the few studies undertaken so far have shown that the disease is a serious constraint to production of pigs kept under intensive indoor systems [2, 3]. However, little is known regarding the prevalence of sarcoptic mange in free-range pigs. 

Hog louse (*Haematopinus suis*) is also a common ectoparasite of pigs all over the world [4–6]. The parasite infests pigs of any age or body condition, although pigs kept outdoors and those in poor body condition are more susceptible [5]. The blood sucking activity of the hog louse results in irritation and discomfort of the pigs and may lead to extensive hair loss and wounds, reduced performance, and increased susceptibility to other diseases [4, 6]. The hog louse has also been implicated in the transmission of diseases such as swine pox, eperythrozoonosis, and African swine fever [7]. 

There is little information on the occurrence of other ectoparasites of free-range pigs in Kenya. Free-range pig keeping is a major activity in western Kenya and in urban areas of the country and is characterized by poor management and low inputs [8]. In a previous questionnaire survey, farmers keeping these pigs indicated that ectoparasites were of major significance, although only a few undertook any control strategy [8]. Data on the epidemiology of various ectoparasites is important in devising an effective control strategy [5]. This study was thus carried out to determine the prevalence of ectoparasites and associated risk factors in pigs kept under free-range conditions in Busia District, Kenya.

## 2. Materials and Methods

### 2.1. Study Area

The study was conducted in Busia District, which is situated in the Western Province of Kenya. The district is located approximately 500 km from Nairobi and lies between latitudes 0°136′ and 0° north of the equator and longitudes 33°54′ and 34°25′ east of Greenwich meridian. The district covers an area of 1261.3 km^2^ and is made up of six administrative divisions, which are Budalangi, Funyula, Matayos, Township, Nambale, and Butula. It lies within the Lake Victoria basin and has an altitude ranging between 1,130 and 1,375 meters above sea level. Most parts of Busia District receive 1270–1790 mm mean annual rainfall, which is bimodal and generally decreases from north to south. The temperature in the district ranges between 14 and 30°C. In 2009, the livestock population was estimated to be 102,377 cattles, 22,449 sheeps, 46,317 goats, and 31,585 pigs [9].

### 2.2. Study Farms

The study was undertaken in pig farms selected from the six administrative divisions of Busia District with the help of extension and administrative officers. Two administrative villages per division were purposively selected based on the presence of higher number of pigs. At village level, households with pigs were established using the snowballing method and sampling to redundancy method [10]. The village chairman identified the first few pig farmers, who helped in identifying the others until all the farmers in the village were covered. To determine the required sample size for pigs, the formula given by Martin et al. [11] was used. A total of 306 pigs from 135 farms were sampled from all the six divisions in Busia District.

### 2.3. Examination for External Parasites and Infestations

All the pig skins were examined for ectoparasites and skin lesions throughout the external surface of the body. Earwax and skin scrapings were examined for the presence of mites as described by Kaufmann [12]. Ticks and lice presence, location, and intensity were recorded, then picked and stored in 70% alcohol for later identification according to the keys provided by Soulsby [13] and Kaufmann [12]. The intensity of lice was arbitrary categorized as light (<10 lice, whole body count) and heavy (10 or more lice, whole body count).

### 2.4. Collection of Data on Pigs, Management Practices, and Rainfall

The host related attributes which accompanied the sampling of each pig included sex and age (category). The categories from which samples were obtained included piglets (23), growers (135), finishers (53), sows (84), and boars (11). In the study, piglets were around two months of age [8]. The growers were regarded as the class of pigs which were already weaned but were less than 16 weeks (4 months) old. The finishers were the pigs aged between 4 and 10 months. Sows were regarded as the breeding females which were either pregnant or had previously farrowed. Boars were few (11) and were excluded from the statistical analysis on relationship between occurrence of parasites and categories. A short structured questionnaire on personal biodata, administrative division, history of acaricides spraying, and housing of pigs was administered to the participating farmers. Since the definition of a division was administrative, the amount of annual rainfall was also considered. 

### 2.5. Statistical analysis

The collected data was entered into Ms Excel (MS Software, Microsoft, USA) spreadsheets before being exported to Statview (SAS Institute Inc., 1995–1998, Cary, NC, USA) package for statistical analyses. Associations between the categorical variables were examined using chi-square (*χ* ^2^) statistic. Pearson correlations (*r*) between the amount of rainfall and prevalence of the parasite were calculated. Multivariate analysis for the most common ectoparasite (*H. suis*) was also undertaken using a backward stepwise analysis to test for association between prevalence of *H. suis* and the independent variables. The level of significance was determined at 95% (*P* value < 0.05), and all tests were two sided.

## 3. Results

### 3.1. Summary of Ectoparasites

The types of ectoparasite infestations observed in the current study included *Haematopinus suis* (hog lice), *Sarcoptes scabiei* (mites), and *Ixodid* spp. (ticks). At animal level, the overall prevalence of *H. suis* (pediculosis) and *S. scabiei* mange was 96.1% (95% CI = 93.9–98.3%) and 63.7% (95% CI = 58.3–69.1), respectively. The percentage of farms infested (with at least one pig infested) with *H. suis* and *S. scabiei* was 99% and 78.7%, respectively. Pigs with heavy infestation (90%) of *H. suis* were more than those with light (10%) infestation. The overall prevalence of ticks was 29.7% (95% CI = 24.6–34.9%), and the observed species included *Rhipicephalus appendiculatus*, *Boophilus decoloratus*, and *Amblyomma variegatum*, in proportions of 70%, 31%, and 12%, respectively. The tick specific-herd prevalence was 46.7% (95% CI = 38.1–5.2%). 

### 3.2. *Haematopinus suis*

All (100%) the pigs originating from Funyula, Butula, Matayos, and Budalangi Divisions were infested with *H. suis* (Table 1). Univariate analysis showed that the prevalences of *H. suis* infestation in these divisions were higher (*P* < 0.05) than those recorded in pigs from Township Division. The prevalence of *H. suis* was negatively correlated (*r* = −0.71) with the amount of rainfall in the respective division of sampling. In descending order, the pigs with heavy *H. suis* infestation were recorded in Nambale (96.8%), Funyula (94.9%), Matayos (91.9%), Butula (90%), Budalangi (83.3%), and Township Divisions (76.6%), and the differences were statistically significant (*χ* ^2^ = 53.3, *P* < 0.05).

The prevalence of *H. suis* in male and female pigs was 96.7% and 94.8%, respectively, and the differences were not statistically significant (*P* > 0.05). In descending order, the prevalence of *H. suis* was highest in finishers (98.1%), growers, (97%), and sows (95.2%) and lowest in and piglets (91.3%), and the differences were not statistically significant (*P* > 0.05). The proportion of pigs with heavy lice infestation was recorded in sows (92.5%), growers (92.4%), finishers (86.5%), piglets (76.2%), and the differences were statistically significant (*χ* = 36.2, *P* < 0.05). Sixty-two percent (62%) of the farmers did not have a history of spraying their pigs with acaricide and those who sprayed did it irregularly. History of acaricide spraying was not significantly (*P* > 0.05) associated with prevalence of *H. suis*. Pigs which were housed (night shelters, 39% of the farms) had significantly (*P* < 0.05) lower prevalence of *H. suis* than those from households without any form of housing.

In the final multivariate model, divisions of origin and housing were the only significant predictors of prevalence of *H. suis*. Thus, pigs from Funyula, Nambale, Butula, and Matayos were 2.5, 1.9, 1.7, and 1.1 more likely to be infested with lice than those from Budalangi. However, pigs from Township Division were 3.5 times less likely to have lice infestation when compared to those from Budalangi. Further, pigs in farms where housing was provided were 2.3 times less likely to have lice infestation than those where housing was provided. 

### 3.3. Sarcoptes scabiei

In descending order, the prevalence of *S. scabiei* (Table 1) was highest in Funyula Division and was lowest in Budalangi Division, and the differences were significant (*P* < 0.05). There was a negative correlation (*r* = −0.78) between the amount of rainfall and prevalence of *S. scabiei*. The prevalence of *S. scabiei* was higher in female (65.6%) than male (59.8%) pigs, but the difference was not statistically significant (*χ* ^2^ = 0.45, *P* > 0.05). In descending order, mange infestations were more common in sows (71.4%), finishers (69.8%), and growers (58.5%) and least in piglets (43.5%). The prevalence of *S. scabiei* was significantly (*P* < 0.05) lower in piglets than finishers and sows. The rest of comparisons were not statistically significant (*P* > 0.05). The prevalence of *S. scabiei* was not significantly (*P* > 0.05) associated with history of spraying and provision of housing.

### 3.4. Ticks

In descending order, the prevalence of ticks' infestation was highest in Nambale Division and lowest in Budalangi Division (Table 2). There were statistically significant (*χ* ^2^ = 18, *P* < 0.05) differences in divisional specific tick prevalence. The prevalences were significantly lower (*P* < 0.005) in pigs from Budalangi and Butula Divisions than those from the other divisions. There was a strong positive correlation (*r* = 0.98, *P* < 0.05) between the prevalence of ticks and the amount of rainfall. The proportion of pigs infested with *Rhipicephalus appendiculatus* was highest in pigs from Matayos Division but lowest in pigs from Nambale Division (Table 2). However, the proportion of pigs infested with *Boophilus decoloratus* and *Amblyomma variegatum* was highest in pigs from Nambale Division. 

There were more male (35.1%) infested with ticks than female pigs (27.3%), although the difference was not statistically significant (*χ* ^2^ = 1.9, *P* > 0.05). In descending order, the ticks prevalence was highest amongst the finishers (35.9%) followed by growers (28.1%), sows (27.4%), and piglets (21.7%); the differences were not statistically significant (*χ* ^2^ = 5.2, *P* > 0.05). The prevalence of ticks was significantly (*P* < 0.05) lower in pigs from farms with housing bomas (night shelters) when compared with those from farms where housing was not provided. 

## 4. Discussion

The ectoparasitic infestations found in pigs in the current study included mange (caused by *S. scabiei*), pediculosis (*H. suis*), and hard ticks. This study constitutes the first major report on occurrence of ectoparasites in free-range pigs in Kenya. The prevalence of *S. scabiei* mange pigs reported in our study was higher than that reported in other studies in Germany (19.1%) [5], Ghana (38.3%) [7], Botswana (40%) [6], and Tanzania (52%) [3] but was lower than that reported in breeding farms in Spain (92.8%) [14]. The cause of high prevalence of sarcoptic mange in pigs from the study area included free-range conditions and lack of ectoparasite control by majority of farmers. The prevalence of mange was related to the age of pigs being highest in sows but lowest in piglets, and this has been previously reported in other countries such as Spain [14]. The higher prevalence of mange in sows could be related to the longer period the sows had been kept in the farm compared to other age groups of pigs. The prevalence of clinical mange varied with divisions, being lowest in the drier Budalangi Division, showing that the survival of sarcoptic mites may be affected by the dry conditions in this division. It will be important to devise an effective control strategy for control of mange in pigs in the extensive system of production, where most of the farmers are resource poor and might not be able to afford the conventional acaricides [9]. 

The overall prevalence of pediculosis was high (96.1%) showing that *H. suis *development and transmission were highly favourable in the study area. The animal prevalence reported in this study was higher than that reported in Germany [5] (2.5%) and Ghana (66.7%) [7] but was of a similar range with that reported in indigenous free-range pigs from Botswana (100%), [6]. Although knowledge on the possible risk factors for transmission of *H. suis* is scanty, pasturing of pigs, purchase of replacements from infected farms, and keeping of pigs in dirty and unhygienic conditions have been indicated to cause an increase in prevalence of hog louse in a farm [5, 6]. All these conditions, together with inadequate ectoparasite control, were evident in the study area, where high prevalence was associated with lack of spraying and housing. The level of infestation was significantly associated with the class of the pigs, with heavy infestation being more common in sows, possibly because the latter had stayed longer in the farms compared to other classes of pigs. It can also be hypothesized that higher levels of prolactin and progesterone as well as stresses of lactation and pregnancy make female animals more susceptible to parasitic infestations [1, 5]. This shows that sows can be a major source of infestation to the piglets. 

Hog louse is a major cause of pruritus and anemia in pigs and has been implicated as a potential vector of several diseases including *Mycoplasma suis* [5, 7], the causative agent of an acute febrile, haemolytic disease in feeder pigs. The suggested transmission of African swine fever by hog louse has, however, not been confirmed, and it would important to determine whether these parasites play any significant role in maintaining the endemicity of ASF in the study area [15]. Further, following a comprehensive epidemiological study, an effective control strategy (targeting mainly housing and spraying) for control of *H. suis* should be devised for the free-range pigs. 

Hard ticks found to be infesting pigs included *Rhipicephalus appendiculatus*, *Boophilus decoloratus*,and *Amblyomma variegatum*. A similar range of species was previously reported in cattle from the same district [16], possibly because they share the same habitat. Literature on occurrence of ticks in pigs is scanty possibly because most studies have been undertaken in indoor pigs, where transmission of ticks is expected to be minimal. However, in extensive systems where pigs are reared in pasture or are scavengers, tick transmission either between pigs or from other livestock is common [17]. The prevalence reported in this study was lower than that reported in free-range pigs in Botswana (100%) [6] and Ghana (58.3%) [7] and wild pigs in USA (99%) [18]. Permin et al. [7] reported three species of ticks (species similar to our study), while Nsoso et al. [6] observed only *Rhipicephalus evertsi evertsi* and *Amblyomma hebraeum*. The tick load in the current study was low and was close to that reported in free-range pigs from Botswana [6] where it ranged from 1.44 ± 0.35 to 2.69 ± 0.35 ticks per animal but was lower than that reported in Brazil (9.6 ticks per animal) [19]. The prevalence of ticks was higher in divisions with high rainfall which are known to favour the survival and fecundity of ticks [20]. Age-related occurrence of ticks was noted in this study and has been reported among the wild pigs in USA, where piglets had lower prevalence than the other groups [18]. Compared to other groups of pigs, the piglets had stayed in the farms for a shorter period and thus could have a less exposure to ticks. Further, pigs which were not provided with housing had higher prevalence of ticks possibly because of poor husbandry associated with lack of housing and spraying of acaricides.

In conclusion, this study has described the occurrence of ectoparasites in free-range pigs in Busia District, Kenya. The observed parasites were related to rainfall pattern, age of pigs, and husbandry factors such as lack of housing and absence of ectoparasites control in most farms. For effective control of these parasites, farmers will require to be educated on proper pig husbandry and usage of acaricides.

## Figures and Tables

**Table 1:** Distribution of division-specific prevalence of *Sarcoptes scabiei* and *Haematopinus suis* in pigs from Busia District, Kenya.

Divisions	*Sarcoptes scabiei *		*Haematopinus suis *	
	Prevalence (%)	95% CI	Prevalence (%)	95% CI
Budalangi	23.3	7.3–39.4	100	100-100
Township	77.2	66–88.4	82.5	72.3–92.6
Butula	77.5	64–91	100	100-100
Funyula	84.6	72.8–96.5	100	100-100
Matayos	54.1	42.4–65.7	100	100-100
Nambale	60.6	48.5–72.7	97	92.7–100
Overall	63.7	58.3–69.1	96.1	93.9–98.3

Key: CI: confidence interval.

**Table 2:** Prevalence of ticks infesting pigs from the six divisions in Busia District.

Division	Prevalence (95% CI)	Proportion (%) relative to total infected		
		*Rhipicephalus appendiculatus *	*Boophilus decoloratus *	*Amblyomma variegatum *
Township	33 (20.7–46)	68	37	0
Nambale	39 (27.3–51.5)	54	46	35
Funyula	31 (15.6–45.9)	75	25	0
Butula	10 (0.3–19.7)	25	25	0
Matayos	37 (25.3–47.7)	93	11	4
Budalangi	10 (0–21.4)	66	0	33
Overall	29.7 (24.6–34.9)	70	31	12

## References

[1] Davies PR (1995). Sarcoptic mange and production performance of swine: a review of the literature and studies of associations between mite infestation, growth rate and measures of mange severity in growing pigs. *Veterinary Parasitology*.

[2] Wabacha JK, Maribei JM, Mulei CM, Kyule MN, Zessin KH, Oluoch-Kosura W (2004). Health and production measures for smallholder pig production in Kikuyu Division, central Kenya. *Preventive Veterinary Medicine*.

[3] Kambarage DM, Msolla P, Falmer-Hansen J (1990). Epidemiological studies of sarcoptic mange in Tanzanian pig herds. *Tropical Animal Health and Production*.

[4] Davis DP, Williams RE (1986). Influence of hog lice, *Haematopinus suis*, on blood components, behavior, weight gain and feed efficiency of pigs. *Veterinary Parasitology*.

[5] Damriyasa IM, Failing K, Volmer R, Zahner H, Bauer C (2004). Prevalence, risk factors and economic importance of infestations with Sarcoptes scabiei and *Haematopinus suis* in sows of pig breeding farms in Hesse, Germany. *Medical and Veterinary Entomology*.

[6] Nsoso SJ, Mannathoko GG, Modise K (2006). Monitoring production, health and marketing of indigenous Tswana pigs in Ramotswa village of Botswana. *Livestock Research for Rural Development*.

[7] Permin A, Yelifari L, Bloch P, Steenhard N, Hansen NP, Nansen P (1999). Parasites in cross-bred pigs in the Upper East Region of Ghana. *Veterinary Parasitology*.

[8] Kagira JM, Kanyari PWN, Maingi N, Githigia SM, Ng’ang’a JC, Karuga JW (2010). Characteristics of the smallholder free-range pig production system in western Kenya. *Tropical Animal Health and Production*.

[9] Government of Kenya (2009). *Kenyan Livestock Population Census*.

[10] Sikasunge CS, Phiri IK, Phiri AM, Dorny P, Siziya S, Willingham AL (2007). Risk factors associated with porcine cysticercosis in selected districts of Eastern and Southern provinces of Zambia. *Veterinary Parasitology*.

[11] Martin SW, Meek AH, Willeberg P (1987). *Veterinary Epidemiology, Principles and Methods*.

[12] Kaufmann J (1996). *Parasitic Infections of Domestic Animals: A Diagnostic Manual*.

[13] Soulsby EJL (1982). *Helminths, Arthropods and Protozoa of Domesticated Animals*.

[14] Alonso de Vega F, Mendez de Vigo J, Ortiz Sanchez J, Martinez-Carrasco Pleite C, Albaladejo Serrano A, Ruiz de Ybañez Carnero MR (1998). Evaluation of the prevalence of sarcoptic mange in slaughtered fattening pigs in southeastern Spain. *Veterinary Parasitology*.

[15] OIE/WAHID ASF outbreak in Kenya.

[16] Karanja SM (2005). *Epidemiology and importance of trypanosomosis, helminthosis and tickborne diseases on performance of cattle in Busia District, Kenya [Ph.D. thesis]*.

[17] Holness DH (2005). *Pigs. The Tropical Agriculturalist*.

[18] Greiner EC, Humphrey PP, Belden RC, Frankenberger WB, Austin DH, Gibbs EP (1984). Ixodid ticks on feral swine in Florida. *Journal of Wildlife Diseases*.

[19] Labruna MB, Camargo LMA, Schumaker TTS, Camargo EP (2002). Parasitism of domestic swine (*Sus scrofa*) by Amblyomma ticks (Acari: Ixodidae) on a farm at Monte Negro, Western Amazon, Brazil. *Journal of Medical Entomology*.

[20] Knopf L, Komoin-Oka C, Betschart B, Jongejan F, Gottstein B, Zinsstag J (2002). Seasonal epidemiology of ticks and aspects of cowdriosis in N’Dama village cattle in the Central Guinea savannah of Côte d’Ivoire. *Preventive Veterinary Medicine*.

